# Cost of resistance to trematodes in freshwater snail populations with low clonal diversity

**DOI:** 10.1186/s12898-017-0152-x

**Published:** 2017-12-13

**Authors:** Yael Dagan, Evsey Kosman, Frida Ben-Ami

**Affiliations:** 1 School of Zoology, George S. Wise Faculty of Life Sciences, Tel Aviv University, 6997801 Tel Aviv, Israel; 2 Institute for Cereal Crops Improvement, Tel Aviv University, 6997801 Tel Aviv, Israel

**Keywords:** Clonal diversity, *Melanoides tuberculata*, Parasitism, Red Queen hypothesis, Trade-offs

## Abstract

**Background:**

The persistence of high genetic variability in natural populations garners considerable interest among ecologists and evolutionary biologists. One proposed hypothesis for the maintenance of high levels of genetic diversity relies on frequency-dependent selection imposed by parasites on host populations (Red Queen hypothesis). A complementary hypothesis suggests that a trade-off between fitness costs associated with tolerance to stress factors and fitness costs associated with resistance to parasites is responsible for the maintenance of host genetic diversity.

**Results:**

The present study investigated whether host resistance to parasites is traded off with tolerance to environmental stress factors (high/low temperatures, high salinity), by comparing populations of the freshwater snail *Melanoides tuberculata* with low vs. high clonal diversity. Since polyclonal populations were found to be more parasitized than populations with low clonal diversity, we expected them to be tolerant to environmental stress factors. We found that clonal diversity explained most of the variation in snail survival under high temperature, thereby suggesting that tolerance to high temperatures of clonally diverse populations is higher than that of populations with low clonal diversity.

**Conclusions:**

Our results suggest that resistance to parasites may come at a cost of reduced tolerance to certain environmental stress factors.

## Background

An organism will invest in defense mechanisms against pathogens and parasites, depending in part on the balance between the advantages of not becoming infected and the costs of maintaining these defenses. As these mechanisms are partly heritable, they can be costly to maintain and express (e.g. constitutive and inducible resistance in plants), but the extent of their use and the actual costs they incur in natural populations is unknown [1]. It has been suggested that this balance, or trade-off between the advantages of being resistant and the costs of defense, is partly responsible for maintaining genetic diversity in natural host populations [2–6]. Particularly in animal host-parasite systems, evidence of these costs and their underlying causes are scarce [7, 8]. For example, Webster and Woolhouse [9] found that resistance and susceptibility to infection are heritable in the *Biomphalaria glabrata*–*Schistosoma mansoni* snail-trematode system. They further showed that susceptible snails were more fertile (number of offspring produced) than resistant-selected or unselected control snail lines.

Although the cost of resistance has been tested extensively in the laboratory using laboratory-maintained lines of hosts and parasites (e.g. [10, 11]), it has rarely been tested using animals from natural populations [12, 13]. Furthermore, it is not always the case that susceptible hosts can cope with environmental stress factors better than resistant hosts [14]. Here we used the freshwater snail *Melanoides tuberculata* (Muller) to investigate whether host resistance to parasites is traded-off with tolerance to environmental stress factors (high/low temperatures, high salinity). The rationale for the present study is an earlier study in which we found that parasite-mediated selection promoted clonal diversity in *M. tuberculata* populations [15]. Furthermore, computer simulations and empirical studies have shown that parasites can select for the accumulation of clonal diversity for resistance [16, 17]. We therefore sampled snails from populations with varying degrees of clonal diversity, as our aim was to compare the stress tolerance of snails originating from natural populations with low vs. high clonal diversity. Given that polyclonal populations were more parasitized than populations with low clonal diversity, we expected them to tolerate environmental stress conditions better than populations with low clonal diversity.

## Methods

### Host-parasite system

The cerithioidean gastropod *Melanoides tuberculata* is native to North Africa and the Middle East [18, 19], but has invaded North and South America, East Africa, Southern Asia and Australia [20]. It is a warm-temperate to tropical freshwater dweller, typically found in shallow slow-running water, especially on soft mud and sand substrata [21, 22]. Female *M. tuberculata* usually reproduce parthenogenetically (obligate apomixis) [23–25], but also via sporadic sexual reproduction in the presence of males [26]. In Israel, the frequency of fertile males can reach up to 66% [21, 27–30]. Furthermore, sex plays a crucial role in the ability of *M. tuberculata* to invade new ecosystems, because it amplifies the effect of multiple introductions of the snail by generating novel trait combinations [31, 32].

*Melanoides tuberculata* is the first intermediate host for several trematodes that have important public health and agricultural implications (e.g. eye fluke; [33–37]). The parasite usually develops parthenogenetically within the snail, sterilizes it, and cercariae liberated from infected snails encyst on the gills of fish. The larval worms become adults after being consumed by waterfowl or waders, wherein they reproduce sexually and their eggs are released with the definitive host’s feces [38]. These eggs hatch into free-swimming miracidia that penetrate snail tissue, thus completing the parasite’s life cycle.

### Sampling and data collection

During the summer of 2011, we collected 120 adult snails each from six *M. tuberculata* sites (populations) and transported them alive to the laboratory. The collection sites included streams, ponds and springs along the Mediterranean Coast, in the Jordan and Beit-She’an Valleys, and in the Judean and Negev Deserts. Three of these sites are known to harbor relatively few clonal lineages (Ein Kaftor, Majrase and Nofarim) and three are known to be polyclonal (Sapir, Timna and Zafzefa), based on the analysis of nine allozyme loci during the same period [15]. Natural infection prevalence in the polyclonal sites ranged from 0 to 57.1%, whereas the sites with low clonal diversity were not parasitized [15]. We also collected annual daily temperature data, based on measurements of the Israeli meteorological service stations proximate to the sampling sites.

### Experimental design

To examine possible trade-offs among environmental stress tolerance, resistance to parasite infection, and clonal diversity, we exposed snails from populations with varying degrees of clonal diversity to three environmental stress factors: high (40 °C) and low (5 °C) temperatures, as these are the maximum and minimum temperatures near the sampling sites during summer and winter, respectively; and high salinity (30 parts per thousand, or ppt), resembling salinity levels ten times greater than those in the sampling site with the highest salinity (Zafzefa). Thirty adult snails from each population were used per treatment. Prior to the experiment all snails were measured and sexed based on the color of their gonads [39]. Additionally, each snail was individually exposed to direct light and screened for infection by trematodes. We excluded the Zafzefa population from subsequent analysis as a result of finding a large proportion of infected snails, which prevented us from determining their cause of death. Uninfected females were transferred each to a separate 100 mL jar, whereas infected females and males were discarded. In total 600 snails were used (five populations × [three stress treatments + controls] × 30 snails per treatment). The salinity treatments and control groups were kept at 25 °C. Snails were fed with *Spirulina* algae powder (1 g in 1 mL) every other day, and water was replaced once a week, high-salinity ventilated water to salinity treatments and ventilated water to all other treatments and control groups. Snail mortality was monitored on a daily basis and upon death, the snail’s gonad and digestive gland were examined under a light microscope to confirm that the cause of death was unrelated to parasitic infection. The experiment was carried out for 153 days, and the jars in each treatment were randomly shuffled on a weekly basis to avoid position effects.

### Clonal diversity analysis

Population diversity was estimated using the Shannon–Wiener and Kosman indices. The index calculations presented herein are based on snails sampled during the same period from the same natural populations. The Shannon–Wiener entropy is based on relative frequencies of different genotypes [40], but the extent of similarity between those genotypes is not taken into account. The Kosman assignment-based diversity, *KW*_*ρ*_, is a more complicated index [41, 42]. It does take into account the contribution of dissimilarity *ρ* among individuals to the diversity within a population and copes with an individual genotype as a fixed combination of possibly associated alleles. The latter is extremely important for studies of populations with probable clonal reproduction.

The degree of dissimilarity among individuals (*ρ*) contributes considerably to the diversity within a population. Therefore, selection of a proper dissimilarity measure is one of the decisive issues in analyzing structure and diversity of populations. Because allozymes are codominant markers, they allow for determining different alleles at each locus, so that the number of those alleles does not exceed the ploidy of an organism. However, in general there is no easy way of reconstructing a precise combination of alleles for heterozygotes of polyploids. In addition, we do not have information about the ploidy level of each snail involved in the experiment. Therefore, taking a more conservative approach, we assumed that two individuals are equally distant or undistinguishable at a given locus if they are represented by different or identical combinations of alleles, respectively. Thus, the simple mismatch index, m, is the most suitable measure of dissimilarity between multilocus allozyme profiles of individuals, and diversity within populations was estimated using the assignment-based diversity, *KW*_*m*_, with regard to the simple mismatch dissimilarity *m* (*ρ* = *m*). For each pair of individuals, if an allele combination at a given locus was missing for one of the individuals, this particular locus was discarded from calculating the simple mismatch dissimilarity for the corresponding pair.

**Fig. 1:**
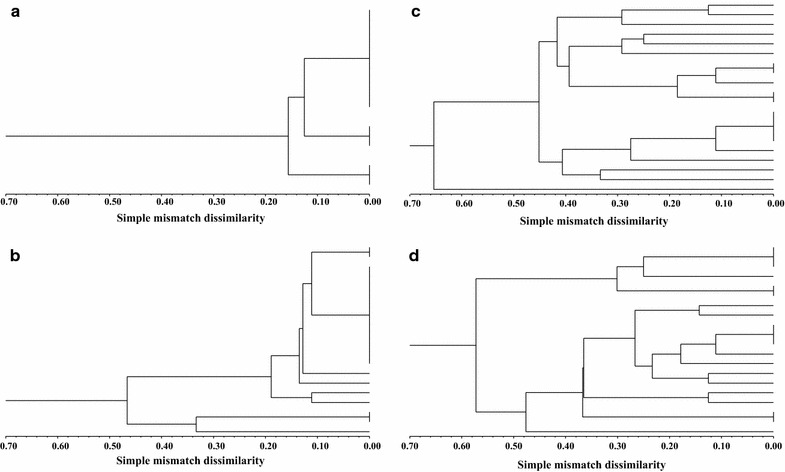
Dendrograms of the populations, showing their clonal diversity (*KW* Kosman index, *NG* number of genotypes, *SW* Shannon–Wiener index): **a** Majrase, *KW* = 0.100, NG = 3, SW = 0.41. **b** Timna, *KW* = 0.222, NG = 8, SW = 0.67. **c** Zafzefa, *KW* = 0.542, NG = 15, SW = 1.12. **d** Sapir, *KW* = 0.564, NG = 14, SW = 1.10. Ein Kaftor (*KW* = 0, NG = 2, SW = 0.30) and Nofarim (*KW* = 0.008, NG = 2, SW = 0.06) did not allow production of relevant dendrograms, as the simple mismatch dissimilarity among individuals was extremely low, as expected from populations with low clonal diversity

Diversity estimates were obtained using the VAT software [43]. Using the unweighted pair group method with arithmetic means (UPGMA), dendrograms for structural relationships between individuals within each population were derived based on the simple mismatch dissimilarity (Fig. 1), using the SAHN module of the NTSYSpc package, v. 2.2 (Exeter Software).

### Statistical analysis

All statistical tests were carried out using GraphPad Prism version 6.01 for Windows (GraphPad Software, http://www.graphpad.com) and Oasis—online application for survival analysis [44]. Kaplan–Meier survival analysis was used to compare among life spans of stress-treated populations, using the log-rank and Gehan–Breslow–Wilcoxon tests. The Gehan–Breslow–Wilcoxon test gives more weight to deaths at early time points, whereas the log-rank test gives equal weight to all time points. We applied both tests to ensure we do not overlook a significant difference between populations with early deaths and populations with more moderate death rates. Bonferroni correction for multiple comparisons was applied when generating the *P* value. We used linear regression to determine the relationship between mean survival (dependent variable) and clonal diversities (Shannon–Wiener and Kosman indices), number of genotypes and average maximum/minimum temperatures in summer/winter as independent variables.

## Results

**Fig. 2:**
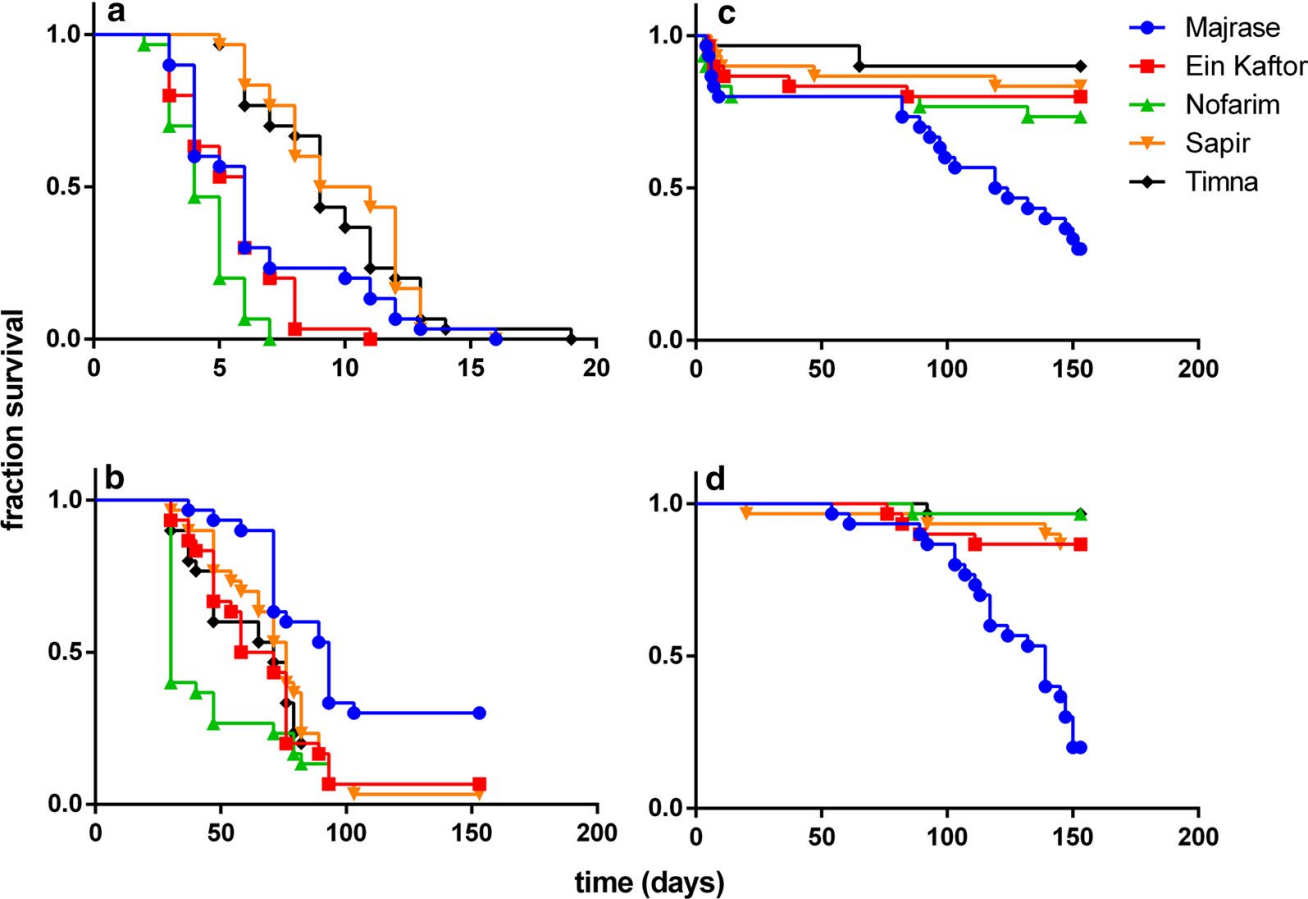
Kaplan-Meier survival curves for five *M. tuberculata* populations under four conditions: **a** high temperature, **b** low temperature, **c** high salinity level, **d** control

We found no differences in the survival of the control groups among four out of five populations (Table 1, Fig. 2d). Only the survival of control snails from Majrase was lower than that of all other control groups. Snails from the five populations differed in their tolerance to high and low temperatures, as well as to high salinity levels (Table 1, Fig. 2a–c). Furthermore, the ranking of tolerance among treatments was not consistent. For example, snails from Sapir and Timna lakes tolerated high temperatures better than snails from Ein Kaftor pond and Majrase stream, which in turn tolerated high temperatures better than snails from Nofarim pool (Table 1, Fig. 2a). Snails from Nofarim were also more sensitive to high salinity levels in comparison with their control group, whereas all other populations were unaffected by this treatment in comparison with their respective control groups (Fig. 2c, d). Snails from Majrase tolerated low temperatures better than snails from Sapir, Ein Kaftor and Timna, which in turn tolerated low temperatures better than snails from Nofarim (Table 1, Fig. 2b).

**Table 1:** Comparison of Kaplan–Meier survival curves for each pair of *M. tuberculata* populations, in all treatments including controls

Population 1	Population 2	High temperature	Low temperature	High salinity level	Control
Majrase	Ein Kaftor	0.1748	*0.0009*	*0.0004*	*<* *1e−04*
Majrase	Nofarim	*0.0014*	*<* *1e−04*	*0.0032*	*<* *1e−04*
Majrase	Sapir	*0.0014*	*0.0011*	*<* *1e−04*	*<* *1e−04*
Majrase	Timna	*0.0064*	*0.001*	*<* *1e−04*	*<* *1e−04*
Ein Kaftor	Nofarim	*0.0041*	0.0015*	0.5272	0.1649
Ein Kaftor	Sapir	*<* *1e−04*	0.5767	0.7197	0.9689
Ein Kaftor	Timna	*<* *1e−04*	0.9046	0.2714	0.16
Nofarim	Sapir	*<* *1e−04*	0.0002*	0.3259	0.1697
Nofarim	Timna	*<* *1e−04*	0.0027*	0.0909	0.9904
Sapir	Timna	0.8041	0.6424	0.4429	0.1658
Majrase	Majrase control	*<* *1e−04*	0.019*	0.9809	
Ein Kaftor	Ein Kaftor control	*<* *1e−04*	*<* *1e−04*	0.4248	
Nofarim	Nofarim control	*<* *1e−04*	*<* *1e−04*	*0.0112*	
Sapir	Sapir control	*<* *1e−04*	*<* *1e−04*	0.6581	
Timna	Timna control	*<* *1e−04*	*<* *1e−04*	0.2945	

We carried out the log-rank (Mantel-Cox) and Gehan–Breslow–Wilcoxon tests. Significant log-rank *P* values are marked in italic and significant Gehan–Breslow–Wilcoxon *P* values are marked with an asterisk

**Fig. 3:**
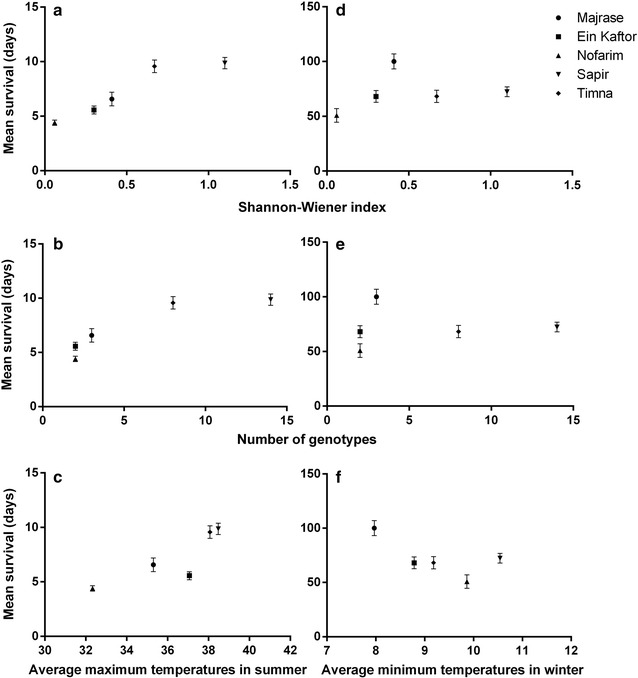
Variation of mean snail survival under high temperature as a function of **a** clonal diversity (Shannon–Wiener index), **b** number of genotypes, **c** average maximum temperature during summer (°C). Variation of mean snail survival under low temperature as a function of **d** clonal diversity (Shannon–Wiener index), **e** number of genotypes, **f** average minimum temperature during winter (°C). Error bars are s.e.m

Clonal diversity (Shannon–Wiener index and number of genotypes) of the populations explained 81–88% of the variation in snail survival under high temperature conditions (Table 2, Fig. 3a, b), whereas the average maximum temperature in each site during the summer could not explain this variation (Table 2, Fig. 3c). Similar results for high temperature conditions, albeit marginally significant, were obtained using the Kosman index (74% of the variation explained, *P* = 0.062). Moreover, under low temperature conditions, clonal diversity (Shannon–Wiener index and number of genotypes) could not explain the variation in snail survival (Table 2, Fig. 3d, e; Kosman index: only 1.6% of the variation explained, *P* = 0.841), and neither could average minimum temperature in each site during the winter (Table 2, Fig. 3f). The latter regressions were not performed on the high salinity survival data, because there was no difference in the tolerance to high salinity among the populations, except for Majrase (Table 1, Fig. 2c). Additionally, in all populations except Nofarim, the survival of control snails did not differ from the survival of snails exposed to the respective high salinity treatment (Table 1).

**Table 2:** Regression analysis showing the variation of mean snail survival under high and low temperatures as a function of clonal diversity (Shannon–Wiener index and number of genotypes), and average maximum/minimum temperatures during summer/winter, respectively

Independent variable	Dependent variable	Condition	*Df*	*F*	*P*	*R*	*R* ^*2*^
Shannon–Wiener index	Mean survival	High temp	4	21.87	*0.0185*	0.9378	0.8794
Number of genotypes	Mean survival	High temp	4	12.99	*0.0366*	0.9013	0.8124
Average max temp during summer	Mean survival	High temp	4	7.186	0.0750	0.8399	0.7055
Shannon–Wiener index	Mean survival	Low temp	4	0.2054	0.6812	0.2532	0.0641
Number of genotypes	Mean survival	Low temp	4	0.0028	0.9610	0.03	0.0009
Average min temp during winter	Mean survival	Low temp	4	2.318	0.2253	− 0.6602	0.4359

Italic typeface indicates a significant effect

## Discussion

The objective of this study was to compare the stress tolerance of snails originating from natural populations with varying degrees of clonal diversity, to determine if there is a cost for being resistant to parasites, in the form of reduced tolerance to environmental stress conditions. We previously found that parasite-mediated selection can promote clonal diversity in *M. tuberculata* populations [15]. Here we performed a stress-manipulating experiment to assess if snails from populations with low clonal diversity, which were found to be less parasitized in our earlier survey, are more sensitive to environmental stress conditions than snails from polyclonal populations, which were found to be more parasitized. Put differently, polyclonal populations were on average less resistant to parasites than populations with low clonal diversity. We expected polyclonal populations to tolerate environmental stress conditions better than populations with low clonal diversity.

Our results demonstrate that tolerance to environmental stress varied considerably across *M. tuberculata* populations. More precisely, about 80 and 70% of the pairs of populations in the high and low temperature treatments, respectively, were significantly distinguishable (Table 1). Such differential tolerance is usually attributed to heritable genetic variation, non-genetic maternal effects or developmental plasticity that contribute to phenotypic variation. Given that sexual and asexual *M. tuberculata* can coexist in natural populations [26], disentangling between genetic and non-genetic variation requires genotyping the snails to determine whether they were produced through outcrossing or parthenogenetically [45]. However, in this study three of the six populations are known to harbor very few distinct clones, i.e. low clonal diversity [15], and even in these three populations, either two out of three or all three pairwise comparisons were statistically significant. Therefore, it is reasonable to assume that the observed variation in tolerance to stress factors has a genetic basis. Given that clonal (genetic) variation in this system is driven at least in part by parasitism [15], we conjecture that resistance to parasitic infections may have contributed to the observed variation in tolerance, insofar that populations with low clonal diversity were less tolerant to environmental stress.

Heat stress can generate lethal reaction in all organisms and it is sufficient to cause cellular damage. Heat stress triggers a heat-shock response, i.e. the enhanced expression of a group of molecular chaperones, collectively called heat-shock proteins (HSPs). Because HSPs prevent the aggregation of heat-damaged proteins and facilitate their renaturation following a heat shock, they are likely to play an important role in thermotolerance [46, 47]. A study of marine snails (genus *Tegula*) from different thermal habitats indicated that there are differences in heat shock responses between snail species that reflect the separate evolutionary histories of these species [48]. These differences may play an important role in setting their thermal tolerance limits and, thereby, their biogeographic distribution patterns. Our results in the high temperature stress experiment, where we found both variation among populations in the reaction to heat stress and a significant correlation with clonal diversity, suggest that high temperature tolerance may be an important trait for *M. tuberculata* adapting to changing environments and coevolving with parasites.

We did not find differences in the tolerance to high salinity among the populations, except for Majrase, which differed from all other populations (Table 1, Fig. 2c). Moreover, in all populations except for Nofarim, the survival of control snails did not differ from the survival of snails exposed to the respective high salinity treatment (Table 1). This may suggest that salinity tolerance per se might not be a good discriminator among populations. Alternatively, it may be that the salinity level we chose—30 ppt—was too low, because close to 80% of the snails in the other four populations survived throughout the experiment. Several studies have shown similar results, i.e. *M. tuberculata* could tolerate high salinities (> 30 ppt) [49–52], albeit a recent study of salinity tolerance in *M. tuberculata* found that salinity levels similar to those used in our experiment had substantial effects on snail mortality [53]. Jacobsen and Forbes [54] found that a gradient of salinity up to 15 ppt can influence life-history traits and feeding rates in the gastropod *Potamopyrgus antipodarum*. Furthermore, approximately fourfold lower salinity levels (8 ppt) were sufficient to reduce the survival of *Melanopsis* spp., the most common freshwater snail genus in Israel [14]. Therefore, future studies should expand the range of salinity levels being tested, in order to identify possible differences in salinity tolerance among *M. tuberculata* populations with different genetic backgrounds.

## Conclusions

The main finding of this study is the existence of variation in environmental stress tolerance among populations with different levels of genetic diversity, insofar that clonally diverse populations can tolerate certain environmental stress factors better than populations with low clonal diversity. Specifically, there appears to be a cost associated with resistance to parasites in *M. tuberculata*, in the form of reduced thermotolerance, which may contribute to explain variation in genetic diversity in these host populations.
